# Association of polymorphisms in leptin receptor gene with obesity and type 2 diabetes in the local population of Coimbatore

**DOI:** 10.4103/0971-6866.69350

**Published:** 2010

**Authors:** Devi Murugesan, Thirunavukkarasu Arunachalam, Viraragavan Ramamurthy, Selvi Subramanian

**Affiliations:** Department of Biotechnology, PSG College of Technology, Coimbatore – 641 004, India

**Keywords:** Allele frequency, case–control study, LEPR polymorphism, local population, obesity, type 2 diabetes

## Abstract

**BACKGROUND:**

Candidate gene association studies are very relevant to the area of clinical pharmacology. As information on candidate genes and candidate single nucleotide polymorphisms increases, a number of such candidates can be studied in a population to explore their association with their susceptible disease. One such attractive and popular Single Nucleotide Polymorphism (SNP) candidate for obesity is the gene coding for leptin receptor. The leptin receptor gene (LEPR) polymorphism plays an important role in obesity and type 2 diabetes. But the role of this polymorphism is not yet studied in Indian population. Hence, the study focused to explore the association of leptin receptor polymorphisms (K109R, Q223R and K656N) with obesity and type 2 diabetes in both diabetic and non-diabetic subjects recruited from the local population of Coimbatore.

**MATERIALS AND METHODS:**

Genotypic analysis for the three polymorphisms has been made for 300 subjects (150 diabetic and 150 non-diabetic) with the age range of 40–60 years using conventional Polymerase chain reaction (PCR)-restriction fragment length polymorphism (RFLP) techniques in a case–control fashion. Allele frequencies were estimated based on the gene count method. Correlation was made with phenotypic variables including body mass index (BMI), waist-to-hip ratio (WHR), insulin and leptin levels for those polymorphisms.

**RESULTS AND CONCLUSION:**

Among the polymorphisms tested in this study, significant association with BMI (*P* < 0.05), WHR (*P* < 0.05) leptin (*P* < 0.001) and insulin (*P* < 0.0001) was observed for the SNP Q223R, whereas in the case of the other two polymorphisms the association was not statistically significant. The significance value was calculated based on the χ^2^ test. The controls are also found to have a higher frequency of homozygous mutants for Q223R and are significantly associated with obesity. These findings support the hypothesis that Q223R polymorphism is associated with obesity. It can be speculated that the controls showing the same allele may develop Type 2 diabetes at a later stage and Q223R can act as a strong marker.

## Introduction

Obesity is a common condition in industrialized societies and is increasing rapidly. Its etiology is complex and results from combined effects of genes, environment, lifestyle, and their interactions.[1,2] Obesity has become a major issue because of its links to type 2 diabetes, hypertension, dyslipidemia, and insulin resistance syndrome.[3] Data emerging over the past several years have shown a worldwide increase in the number of obese people.[4] Bray 2003[5] has defined obesity based on the anthropometric measures such as height, weight, and waist circumference. Accordingly, subjects are categorized as overweight if their body mass index (BMI), which is weight in kilograms divided by height in meters squared, is equal to or greater than 25 kg/m^2^ and obese if their BMI is 30 kg/m^2^ or more (i.e., weight exceeding 20% of the ideal weight), according to the indications of World Health Organization (WHO). Excessive body weight is associated with various diseases; as a result, obesity has been found to reduce the life expectancy.[6] Central obesity is characterized by its high waist-to-hip (WHR) ratio. It is an important risk factor for metabolic syndrome. Metabolic syndrome is a combination of medical disorders which often includes type 2 diabetes mellitus, high blood pressure, high blood cholesterol, and triglyceride levels.[7] The strength of the link between obesity and specific conditions varies. One of the strongest is the link with type 2 diabetes, which is primarily characterized by insulin resistance. Excess weight is the reason behind 64% of cases of diabetes in men and 77% in women.[8]

About 118 candidate genes are so far associated with obesity.[9] Some of the important candidate genes involved in causing obesity are the genes encoding leptin (*LEP*), leptin receptor (*LEPR*), melanocortin 4 receptor (*MC4R*), adiponectin (*ADIPOQ*), corticotrophin releasing hormone1 (*CRHR1*), prohormone convertase1 (*PC1*), pro-opiomelanocortin (*POMC*), and resistin (*RETN*).[10] Among them, leptin and its receptor play the central role. Leptin, encoded by the obesity (*LEP*) gene, is expressed mainly in adipocytes. Their levels are highly dependent on presence of fats in the cell. It is shown to regulate satiety, energy expenditure, neuroendocrine function, and reproductive competence.[11] The biologic activities of leptin on target tissues are carried out through selective binding to a specific receptor, *LEPR*. *LEPR* maps in humans to the 1p31 chromosome and has at least five isoforms. The structure of the leptin receptor is similar to that of the helical cytokine receptor (class I). Leptin receptors form homodimers, which are capable of activating Janus kinases. The Janus kinase is then able to start activators of transcription. Leptin signaling via the Janus kinases and activation of transcription system is largely associated with the long form (*LEPR1*) of leptin receptor.[12] Studies performed on mice showed that the *LEPR1* is important for transmitting the leptin signal to the cells and is located predominantly in the hypothalamus and not in other tissues.[13] However, the short forms are expressed throughout the body, especially in the kidney, lungs, and choroid plexus.[14] Several polymorphisms are commonly occurring in *LEPR* gene, which cause either synonymous or non synonymous substitutions. The role of homozygosity for inactivating mutations of the leptin receptor (*LEPR*) in producing extreme obesity syndromes in laboratory animals is established.[15] Additionally, a small number of extremely obese humans from consanguineous pedigrees have been identified, who are obese due to homozygosity for inactivating mutations of *LEPR*.[16] Heterozygosity for *LEPR* mutations in mice and rats also results in increase in fat stores.[17,18] The question of whether more common polymorphisms of the *LEPR* gene confer increased susceptibility to obesity and its associated morbid disorder type 2 diabetes remains open in South Indian population.

## Materials and Methods

### Study population

The present study included 300 individuals including 150 non-diabetics as control and 150 diabetics aged between 30 and 60 years as case, recruited from PSG Hospitals, Coimbatore. The participant’s age ranges between 41 and 59 years. The mean BMI and WHR values showed no significant difference between subjects and controls. The insulin and leptin levels were found to be higher in subjects than in controls. The subjects answered a questionnaire regarding health, dietary pattern, family history of diabetes and obesity, lifestyle features including physical activity, stress pattern, smoking habit, and consumption of alcohol. Plasma concentrations of leptin and insulin were measured using a commercial direct enzyme-linked immunosorbent assay (ELISA) human leptin kit and Human Insulin kit, respectively, according to the procedure provided by the manufacturer (Linco Research, Inc, St. Louis, MO, USA). Anthropometric measurements, including BMI and WHR, were also calculated. BMI is used to reflect the total body fat, while WHR is an indirect measurement of body fat centralization.

### Genotyping

Five milliliters of venous blood was drawn from each of the individuals and genomic DNA was isolated by salting out procedure.[19] Polymerase chain reaction (PCR)-restriction fragment length polymorphism (RFLP) based genotyping was carried out using gene-specific primers. The primer to amplify the Q223R region was designed using the integrated DNA technology (IDT) tool, 5’ GGCCTGAAGTGTTAGAAGAT 3’ (forward) and 5’ CTGCTCTCTGAGGTGGGAAC 3’ (reverse). For the other two sites, the primer information was taken from Gotoda et al.[20] The amplified products were restricted with the three specific restriction endonucleases, *HaeIII, MspI*, and *BstUI* for K109R, Q223R, and K656N polymorphisms, respectively. For K109R polymorphism, the *HaeIII* produced two fragments for homozygous allele which were of 32 and 68 bp size. For Q223R, *MspI* site was created for its variant allele and it produced the fragments of 173 and 469 bp, which was visualized in 2% agarose gel. For the homozygous K656N polymorphism, 32 and 38 bp size products were produced, which was visualized in 3.5% agarose gel.

### Statistical analysis

A χ^2^ test was performed for genotypic frequencies and to make case–control comparisons for each of the polymorphisms tested using the Graphpad Instat software (Graphpad soft ware, San Diego, CA, USA). Analysis of variance was performed to compare the phenotypic variables of the different genotypes.

## Results

Genotype frequency indicates the most or least prevalent genotype in a population. The genotypic distribution of the three polymorphisms tested in the LEPR gene for obesity and type 2 diabetes is listed in Table 1. Of the three polymorphisms tested, the frequency of occurrence of R allele in the homozygous form of Q223R was significantly higher in both case and control population (53 and 22%, respectively) than the other polymorphisms, K109R, K656N.

**Table 1:** Genotype distribution of the three polymorphisms in the *LEPR* gene

Genotype	Polymorphism					
	K109R		Q223R		K656N	
	Case (n = 150)	Control (n = 150)	Case (n = 150)	Control (n = 150)	Case (n = 150)	Control (n = 150)
W/W	0.67(100)	0.61(91)	0.20(30)	0.49(73)	0.59(89)	0.57(86)
W/M	0.27(40)	0.32(48)	0.45(67)	0.36(55)	0.35(52)	0.34(51)
M/M	0.06(10)	0.07(10)	0.35(53)	0.15(22)	0.06(9)	0.09(13)

W = wild; M = mutant

Since the study is aiming to associate the SNPs with obesity, the case population has been classified as BMI < 25, BMI > 25 < 30 and BMI > 30, on the basis of WHO classification for Asian population. Also, the case has been classified as codominant, dominant, and recessive models (Hardy-Weinberg model) to reveal the influence of genotypes over the body fat mass by subjecting them to χ^2^ analysis. Analysis of variance of phenotypic variables such as BMI, WHR, leptin, and insulin for three polymorphisms is shown in Table 2. It clearly shows that only for Q223R polymorphism, the levels of leptin, insulin, BMI, and WHR meets the statistical significance. The levels of BMI, leptin, and insulin were found to be increased for the QR (26.6 ± 2.65, 0.91 ± 0.09, 25.13 ± 16.92, 27.37 ± 9.68, respectively) and RR (29.8 ± 2.88, 1.05 ± 0.12, 46.09 ± 22.1, 31.23 ± 10.1, respectively) genotype in the case population.

**Table 2:** Genotype phenotype correlation for the three polymorphisms in the *LEPR* gene

Genotype (*N* = 300)		Phenotype											
		BMI			WHR			Leptin (ng/ml)			Insulin (µU/ml)		
		Case	Control	*P*	Case	Control	*P*	Case	Control	*P*	Case	Control	*P*
K109R													
KK	191	24.93 ± 4.85	24.9 ± 3.01	0.95	0.89 ± 0.11	0.86 ± 0.11	0.31	23.67 ± 12.6	17.63 ± 10.44	0.042	18.86 ± 2.9	15.75 ± 5.2	0.1
KR	89	25.86 ± 3.21	24.1 ± 4.01	0.24	0.9 ± 0.12	0.86 ± 0.13	0.45	18.58 ± 8.79	11.21 ± 5.78	0.12	21.56 ± 5.9	18.23 ± 4.3	0.3
RR	20	32.77 ± 4.65	25.9 ± 5.69	0.31	0.97 ± 0.19	0.92 ± 0.14	0.24	32.14 ± 5.69	19.12 ± 11.36	0.32	20.12 ± 7.5	14.52 ± 6.9	0.28
Q223R													
QQ	103	23.1 ± 2.01	22.8 ± 1.69	0.29	0.88 ± 0.06	0.86 ± 0.10	0.32	19.51 ± 5.63	9.14 ± 6.96	0.361	20.81 ± 6.1	16.92 ± 3.6	0.14
QR	122	26.6 ± 2.65	23.8 ± 3.42	0.03	0.91 ± 0.09	0.88 ± 0.14	0.04	25.13 ± 16.92	13.54 ± 4.96	0.0029	27.37 ± 9.68	17.65 ± 5.4	0.0007
RR	75	29.8 ± 2.88	26.4 ± 3.91	0.04	1.05 ± 0.12	0.98 ± 0.10	0.03	46.09 ± 22.1	21.23 ± 5.21	0.0048	31.23 ± 10.1	13.96 ± 6.9	0.0001
K656N													
KK	175	25.7 ± 4.56	24.9 ± 2.98	0.23	0.90 ± 0.06	0.86 ± 0.14	0.29	29.54 ± 12.14	23.44 ± 11.36	0.062	22.97 ± 7.2	20.61 ± 6.4	0.09
KN	103	25.9 ± 3.89	23.45 ± 4.79	0.26	0.89 ± 0.11	0.86 ± 0.11	0.48	27.22 ± 17.64	18.83 ± 12.01	0.201	22.98 ± 9.5	17.02 ± 5.32	0.18
NN	22	26.3 ± 3.65	28.35 ± 3.65	0.15	0.96 ± 0.14	0.93 ± 0.13	0.63	29.34 ± 11.32	20.85 ± 12.63	0.19	23.12 ± 11.2	17.93 ± 9.65	0.16

The association of K109R polymorphism with phenotypic variable BMI showed comparatively higher levels among the case population but no statistical significance was obtained when compared with the control since 83% of the case were either overweight or obese. The same results were observed for WHR also, which is considered to be another indicator of obesity. Similarly, no significant difference was observed among the case and control population with regard to their leptin values. Hyperinsulinemia is one of the characteristic features for type 2 diabetes; hence, the case population showed higher insulin values than the control population but it was not statistically significant. Among the three genotypes, no significant differences were observed for all the phenotypical variables. Hence, it can be concluded that K109R cannot be associated with the obesity and type2 diabetes in the studied local population.

The association of Q223R with phenotypic variable BMI levels was higher for the mutant forms in both the case and control population and hence it can be concluded that the both heterozygous and homozygous variants were highly associated with the BMI levels. Similar results were also obtained for WHR. Higher leptin levels were confined to the variants of Q223R, which confirms that the QR and RR are highly associated with obesity, since the leptin resistance is the prime characteristic of the obesity. Among the case population, higher insulin values were observed for both the variants of Q223R. From this, we can associate the variants of this candidate SNP with the insulin level in the local population studied.

The association of K656N with phenotypic variable BMI levels showed slightly higher among the case population but no statistical significance was obtained when compared with the control since 83% of the case individuals were either overweight or obese. The same results were obtained for WHR also, which is considered to be another indicator of obesity. Similarly, no significant difference was observed among the case and control population for their leptin values. Hyperinsulinemia is one of the characteristic features for type 2 diabetes; hence, the case population showed higher insulin values than the control population but it did not meet the statistical significance. Among the three genotypes, no significant differences were observed for all the phenotypical variables.

## Discussion

This study reports the relationship of leptin receptor polymorphisms on obesity and type 2 diabetes for the first time in the local population. There was a highly significant correlation between leptin receptor polymorphism and the BMI. Frequency of allele R in the homozygous form of Q223R polymorphism was found to be higher (53%) when compared to the other polymorphisms, K109R and K656N, in genotypic distribution [Table 1]. Our result follows that of the previous studies that the frequency of RR allele was found to be higher in different ethnic groups.[21,22] The genotypic distributions for the three exonic polymorphisms were compared using χ^2^ test between the subgroups of subjects and controls. The genotypic distribution of the Q223R polymorphism alone showed significant difference between normal weight, overweight, and the obese subjects, whereas the other two polymorphisms did not show any statistical significance. Analysis of variance of phenotypic variables for the K109R, Q223R, and K656N polymorphisms was done. Despite being the candidate polymorphisms for *LEPR*, K109R and K656N polymorphisms failed to produce statistical significance among subjects and controls in this study. Similar result was obtained for these polymorphisms in Australian and Japanese population in that no significant differences in allele frequency or genotype distribution were observed between normal weight and six overweight (obese) subjects.[21,23] For Q223R polymorphisms, the levels of leptin, insulin, and BMI were found to be significantly increased for homozygous and heterozygous mutants and showed significant difference among the subjects and controls. Evidence of a significant effect of the Q223R polymorphism on obesity and phenotypic variables has also been reported earlier.[24] Hence, it was concluded that the presence of R223 allele in the homozygous form is a significant predictor of leptin, insulin, and BMI levels. Also, it was found that RR homozygous form was significantly associated with type 2 diabetes in the studied population. It can be speculated that the polymorphism Q223R may act as a strong marker in the local population of Coimbatore.
